# Multilevel optimisation of anaerobic ethyl acetate production in engineered *Escherichia coli*

**DOI:** 10.1186/s13068-020-01703-1

**Published:** 2020-04-07

**Authors:** Anna C. Bohnenkamp, Aleksander J. Kruis, Astrid E. Mars, Rene H. Wijffels, John van der Oost, Servé W. M. Kengen, Ruud A. Weusthuis

**Affiliations:** 1 Bioprocess Engineering, Wageningen University and Research, Droevendaalsesteeg 1, 6708 PB Wageningen, The Netherlands; 2 Laboratory of Microbiology, Wageningen University and Research, Stippeneng 4, 6708 WE Wageningen, The Netherlands; 3 Biobased Products, Wageningen University and Research, Bornse Weilanden 9, 6708 WG Wageningen, The Netherlands; 4 Faculty of Biosciences and Aquaculture, Nord University, 8049 Bodø, Norway

**Keywords:** Eat1, Anaerobic, Alcohol acetyl transferase (AAT), *Escherichia coli*, Ethyl acetate, Bioreactor, Fermentation

## Abstract

**Background:**

Ethyl acetate is a widely used industrial solvent that is currently produced by chemical conversions from fossil resources. Several yeast species are able to convert sugars to ethyl acetate under aerobic conditions. However, performing ethyl acetate synthesis anaerobically may result in enhanced production efficiency, making the process economically more viable.

**Results:**

We engineered an *E. coli* strain that is able to convert glucose to ethyl acetate as the main fermentation product under anaerobic conditions. The key enzyme of the pathway is an alcohol acetyltransferase (AAT) that catalyses the formation of ethyl acetate from acetyl-CoA and ethanol. To select a suitable AAT, the ethyl acetate-forming capacities of Atf1 from *Saccharomyces cerevisiae,* Eat1 from *Kluyveromyces marxianus* and Eat1 from *Wickerhamomyces anomalus* were compared. Heterologous expression of the AAT-encoding genes under control of the inducible LacI/*T7* and XylS/*Pm* promoters allowed optimisation of their expression levels.

**Conclusion:**

Engineering efforts on protein and fermentation level resulted in an *E. coli* strain that anaerobically produced 42.8 mM (3.8 g/L) ethyl acetate from glucose with an unprecedented efficiency, i.e. 0.48 C-mol/C-mol or 72% of the maximum pathway yield.

## Background

Ethyl acetate is used on a large scale as an industrial solvent for the production of paints, coatings and resins [30], as well as in the flavours and fragrances industry [9, 19]. The global production of the ester was estimated at 3.5 million tonnes in 2015 [42]. Currently, ethyl acetate is produced from petrochemicals in energy intensive and unsustainable processes. Traditional Fischer Speier esterification makes use of equilibrium reactions and energy used for elevated temperatures and continuous water removal is adding to the costs [10, 24, 16]. A sustainable alternative is the use of biobased processes in which yeasts produce ethyl acetate from sugars or ethanol at high yields [1, 11, 26]. The most prominent and well-studied yeast is *Kluyveromyces marxianus,* which produces ethyl acetate from whey sugars at more than 50% of the maximum pathway yield of 1 mol_ethyl acetate_/mol_glucose_ [27, 44]. Other examples include *Wickerhamomyces anomalus* and *Kluyveromyces lactis* [17].

**Fig. 1:**
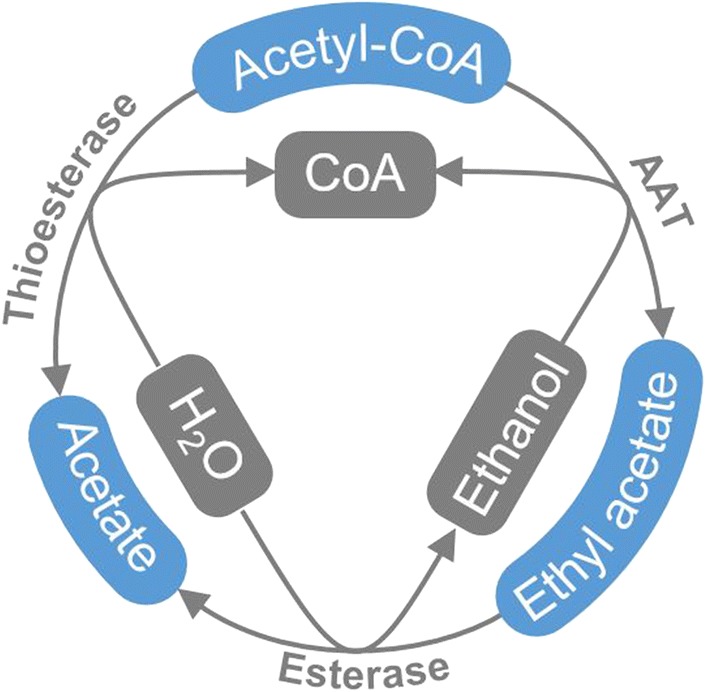
Schematic representation of the three catalytic activities of the Eat1 enzyme for the case ethyl acetate. The AAT activity of Eat1 catalyses the condensation of acetyl-CoA and ethanol into ethyl acetate, ethyl acetate is degraded into ethanol and water as consequence of esterase activity, and acetyl-CoA is converted into acetate, CoA and water exhibiting thioesterase activity

Ethyl acetate production in yeast is catalysed by alcohol acetyltransferases (AATs), which synthesise ethyl acetate from acetyl-CoA and ethanol, releasing free CoA in the reaction [19]. The first-described ethyl acetate-producing AAT was the *Saccharomyces cerevisiae* Alcohol acetyltransferase 1 (Atf1) [33]. However, its homologues in *W. anomalus* and *K. marxianus* appeared to have only a minor role in bulk ethyl acetate production [17, 25]. Instead, they use the recently identified Ethanol acetyltransferase 1 (Eat1) to produce ethyl acetate [17]. Eat1 is targeted to the mitochondria of yeasts due to the presence of an N-terminal mitochondrial pre-sequence [18]. In yeasts, these pre-sequences are cleaved upon arrival in the mitochondrion by cleavage proteins such as mitochondrial-processing peptidase, Oct1 or Icp55 peptidases [34, 47]. Only after all processing events have occurred, the proteins fold into their mature and fully functional form. It has recently been shown that the stability of Eat1, when expressed in *E. coli,* can be improved using N-terminally truncated versions without significantly affecting the specific activity in vitro [20]. An undesirable characteristic of yeast AATs is that they also exhibit thioesterase or esterase side activities, which implies that it is able to hydrolyse acetyl-CoA—the substrate for ethyl acetate production—and also ethyl acetate itself, respectively (Fig. 1). In Eat1, however, these side activities could be subdued by sufficiently high levels of ethanol [17, 35].

**Fig. 2:**
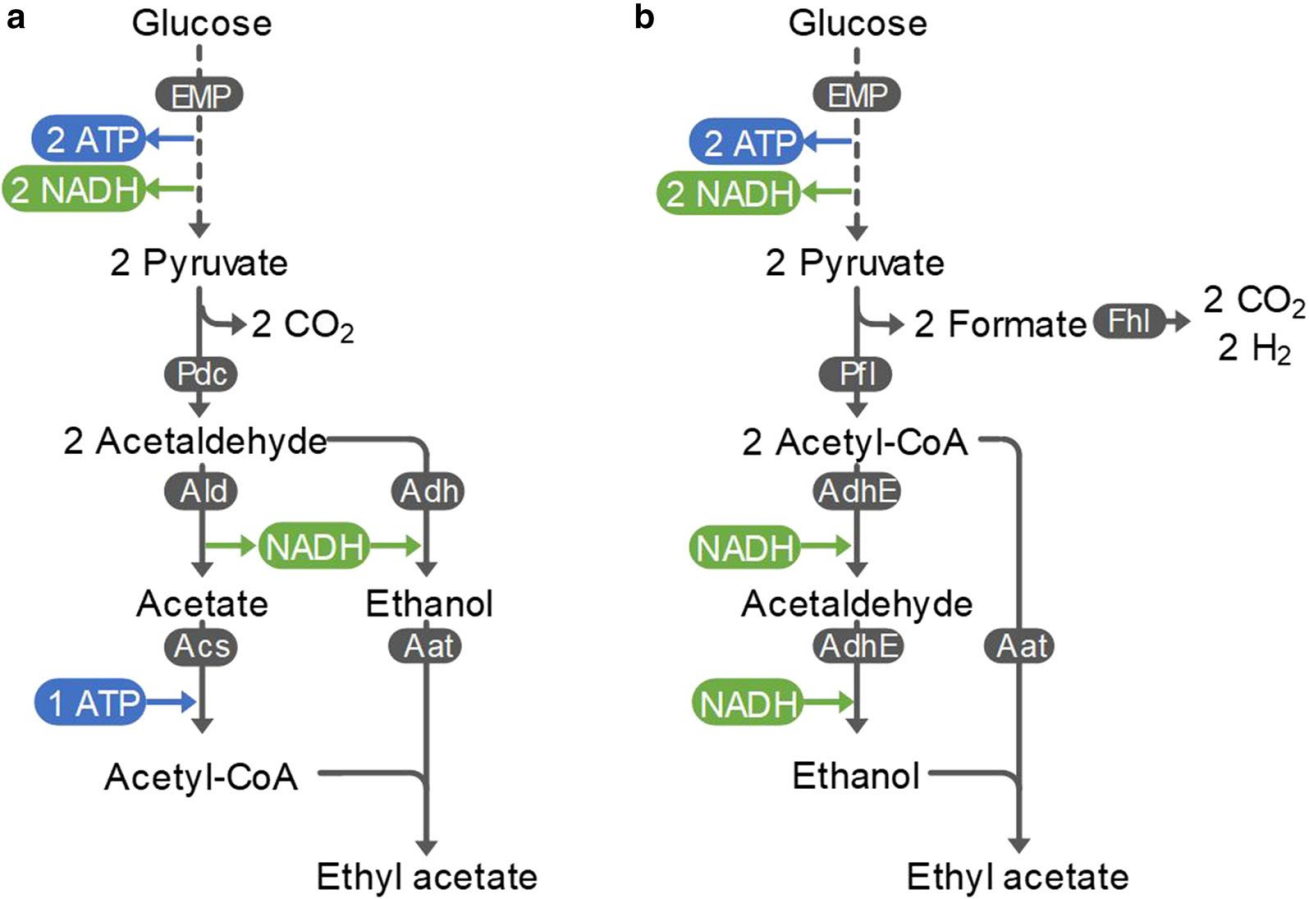
Two anaerobic variants of ethyl acetate production from glucose via the Embden–Meyerhof–Parnas (EMP) pathway. An alcohol acetyltransferase (AAT) catalyses the production of ethyl acetate from acetyl-CoA and ethanol. **a** Ethyl acetate production in yeast. Acetyl-CoA is formed in a series of three reactions: pyruvate decarboxylase (Pdc), acetaldehyde dehydrogenase (Ald) and acetyl-CoA synthetase (Acs). Ethanol is formed from acetaldehyde by an alcohol dehydrogenase (Adh). CO_2_ is produced as a by-product. **b** Ethyl acetate production in bacteria (e.g. *E. coli*). Acetyl-CoA is formed directly from pyruvate via pyruvate formate lyase (Pfl). Ethanol is formed from acetyl-CoA via the bifunctional alcohol/aldehyde dehydrogenase (AdhE). Formate is produced as a by-product that can be converted to CO_2_ and H_2_ via formate-hydrogen lyase (Fhl)

The production of ethyl acetate from glucose results in an NADH surplus [6, 36, 30, 28]. Yeasts are only able to dispose of this surplus by respiration, rendering ethyl acetate production an aerobic process (Fig. 2a). Under these conditions, a significant part of glucose is oxidised in the TCA cycle, leading to lower product yields [50]. Moreover, large-scale aerobic cultivations are often rate limited by the oxygen transfer rate, due to the low solubility of oxygen [13]. Another commonly observed problem in yeast cultivation, particularly in *S. cerevisiae,* is the Crabtree effect, the undesired production of ethanol, consequently lowering the yield on the desired product [8, 27]. *E. coli* and other bacteria can avoid this redox imbalance anaerobically using pyruvate formate lyase (Pfl). Instead of forming NADH, the excess redox equivalents are secreted as formate. In the overall pathway, 1 mol glucose is converted via the EMP pathway to 2 mol pyruvate and 2 mol NADH. Pyruvate is then converted to 2 mol acetyl-CoA and 2 mol formate by Pfl. To maintain the cellular redox balance, the 2 mol NADH produced in glycolysis are regenerated by converting 1 mol acetyl-CoA to ethanol via the bifunctional alcohol/aldehyde dehydrogenase (Adh). A heterologous AAT then condenses the remaining acetyl-CoA with ethanol to form ethyl acetate. This allows redox-neutral production of 0.67 C-mol_ethyl acetate_/C-mol_glucose_ under anaerobic conditions (Fig. 2b). The only by-product of the pathway is formate which can be further converted to CO_2_ and hydrogen by formate-hydrogen lyase (Fhl). The latter can also be considered a valuable product that has potential as a biofuel [40]. The resulting anaerobic process requires less energy for cooling and lower stirring rates due to the absence of transfer limitations, and can be upscaled to larger reactor volumes [4].

A critical step in enabling heterologous ethyl acetate production in *E. coli* is the selection of an efficient AAT catalyst. Both Atf1 and Eat1 have been used to increase ethyl acetate production [38, 17]. AATs from fruit have also been used to evoke ethyl acetate synthesis in *E. coli* under anaerobic conditions and show various affinities for a range of esters [21–23]. However, these enzymes have not yet been compared in the same metabolic background.

In this study, we optimised anaerobic production of ethyl acetate in *Escherichia coli.* We compared and evaluated ethyl acetate production by three AATs from different yeasts after reducing the formation of by-products by creating knockout strains. We optimised gene expression levels using two inducible promotors in combination with several inducer concentrations, and also used N-terminally truncated variants. Final experiments in 1.5-L pH-controlled bioreactors with continuous gas stripping resulted in ethyl acetate production at high yield.

## Results

### Anaerobic ethyl acetate production in *E. coli* and reduction of by-product formation

**Fig. 3:**
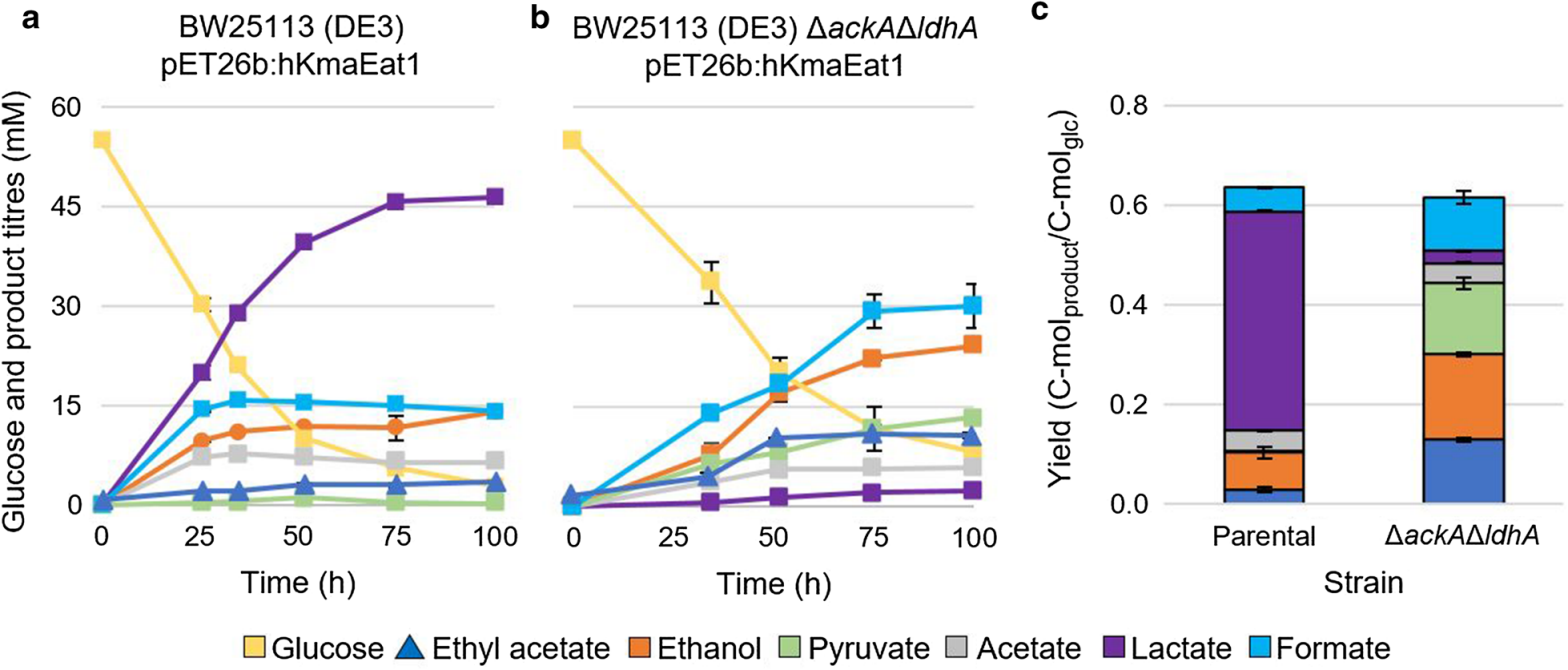
Anaerobic ethyl acetate production in *E. coli* BW25113 (DE3) and *E. coli* BW25113 Δ*ackA*Δ*ldhA* expressing the Kma *eat1* from pET26b:hKmaEat1. **a** Fermentation profile of *E. coli* BW25113 (DE3) (pET26b:hKmaEat1). **b** Fermentation profile of *E. coli* BW25113 Δ*ackA*Δ*ldhA* (DE3) (pET26b:hKmaEat1). **c** Product yields during anaerobic ethyl acetate production. Experiments were performed as biological duplicates. Strains were grown in sealed and N_2_ flushed serum bottles under anaerobic conditions in modified M9 medium at 30 °C and 150 rpm. Gene expression was induced with 0.05 mM IPTG. Error bars represent the standard deviation. Ethyl acetate concentration in the headspace, CO_2_ and H_2_ were not measured

To enable ethyl acetate production in *E. coli* BW25113 (DE3), we introduced the *K. marxianus eat1* (Kma *eat1*) under the control of the IPTG-inducible LacI/*T7* promoter. Under anaerobic conditions the strain produced 2.7 ± 0.1 mM ethyl acetate, representing a yield of 0.03 ± 0.00 C-mol_ethyl acetate_/C-mol_glucose_ (Fig. 3a, c). Due to the formation of by-products, particularly lactate and acetate, the ethyl acetate titre was low. To maximise the metabolic flux towards ethyl acetate, we disrupted the acetate kinase (*ackA*) and lactate dehydrogenase (*ldhA*) genes to reduce acetate and lactate formation, respectively. This increased the ethyl acetate titre to 9.1 ± 0.3 mM (Fig. 3b). The final ethyl acetate yield increased to 0.13 ± 0.00 C-mol_ethyl acetate_/C-mol_glucose_, or 21.4% of the maximum pathway yield (Fig. 3c). Lactate production was almost completely abolished. Acetate yields did not decrease significantly despite the *ackA* disruption (Fig. 3c). A possible explanation is that acetate is produced via the hydrolysis of ethyl acetate or acetyl-CoA by the esterase and thioesterase side activity of eat1, respectively.

Since lactate production can no longer act as sink of NADH, ethanol synthesis should fulfil this role. The conversion of ethanol together with acetyl-CoA to ethyl acetate would basically consume all available NADH and make the entire process redox neutral. However, the accumulation of ethanol and also pyruvate suggests that synthesis of ethyl acetate is limited and that Eat1 activity is the bottleneck of the process (Fig. 3b, c). We therefore focused on optimising the activity of the AAT step.

### Selection of ethyl acetate-producing AAT and gene expression optimisation

**Fig. 4:**
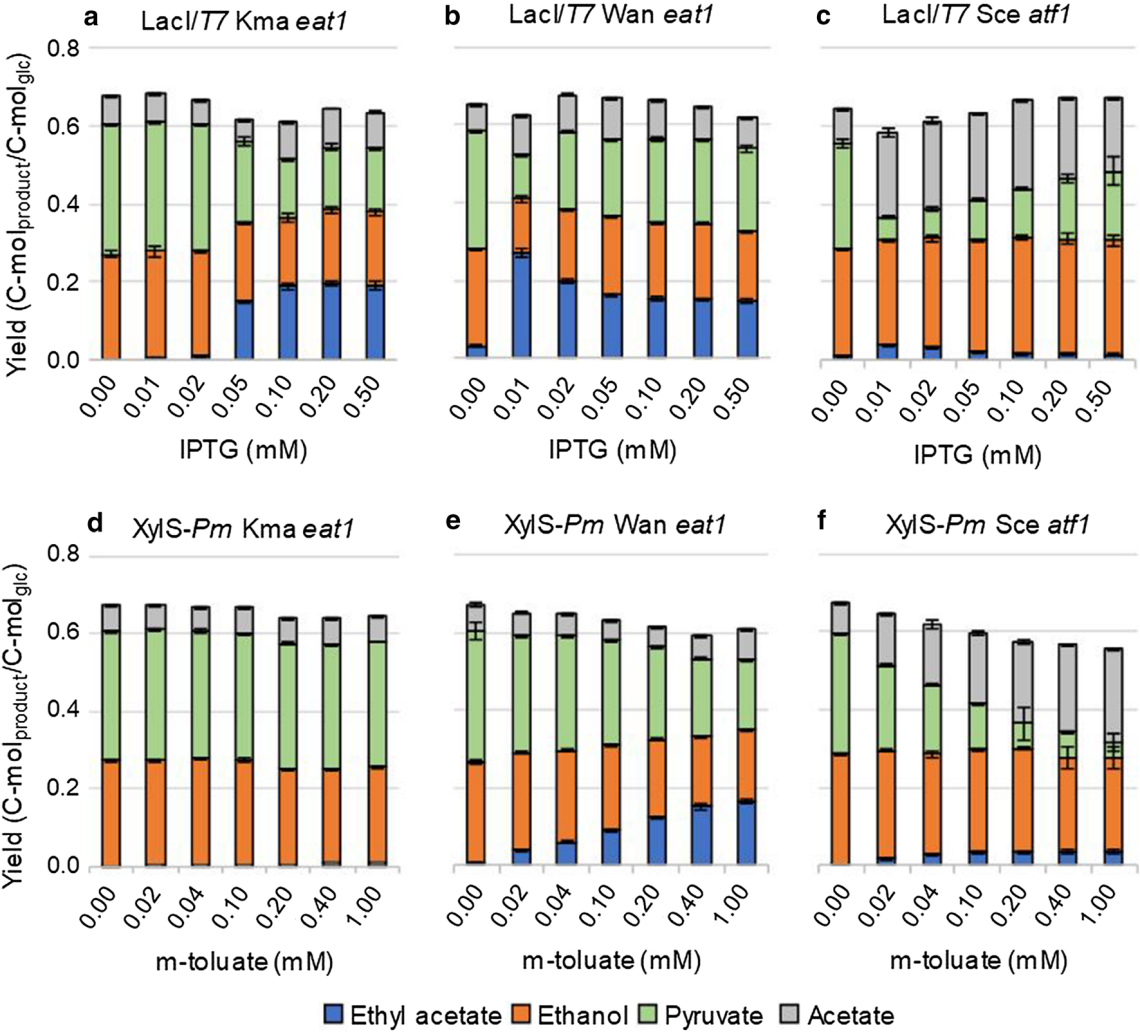
Comparison of three ethyl acetate-producing AAT genes under various gene expression levels, induced by 0.01–0.5 mM IPTG or 0.02–1 mM m-toluate. **a**–**c** Fermentation product yields of strains expressing the Kma *eat1,* the Wan *eat1* and the Sce *atf1*, respectively, under the control of the LacI/*T7* promoter after 120 h of cultivation. Gene expression was induced with 0.01–0.5 mM IPTG. **d**–**f** Fermentation product yields of strains expressing the Kma *eat1,* the Wan *eat1* and the Sce *atf1*, respectively, under the control of the XylS/*Pm* promoter after 120 h of cultivation. Strains were grown in sealed and N_2_ flushed serum bottles under anaerobic conditions in modified M9 medium at 30 °C and 150 rpm. Genes were expressed in *E. coli* BW25113 Δ*ackA*Δ*ldhA* (DE3) from a series of pET26b plasmids. Succinate and formate were detected but are not shown. Experiments were performed as biological duplicates; error bars represent the standard deviation

We compared the ethyl acetate-production capacity of *S. cerevisiae atf1* (Sce *atf1*), Kma *eat1* and *W. anomalus eat1* (Wan *eat1*) genes in *E. coli* BW25113 Δ*ackA*Δ*ldhA* (DE3) cultivated in anaerobic serum bottles. The genes were placed under the control of the inducible LacI/*T7* (Fig. 4a–c) or XylS/*Pm* promoter (Fig. 4d–f) to allow modulation of their expression levels. To induce gene expression, IPTG or m-toluate was added at various concentrations.

Increased AAT activities will reduce the accumulation of pyruvate, increase the production of ethyl acetate when it is active as an AAT, and increase the production of acetate when it is active as either an esterase or thioesterase.

Strains expressing Wan *eat1* (Fig. 4b, e) showed the highest ethyl acetate yields compared to the other AAT genes controlled by the same promoter. High yields of ethyl acetate were also reached by strains expressing Kma *eat1* under control of the lac-*T7* promoter (Fig. 4a). Surprisingly, strains expressing Kma *eat1* under the XylS/*Pm* promoter produced only traces of ethyl acetate under all induction levels (Fig. 4d). Sce *atf1* also evoked ethyl acetate production, but the yields were significantly lower compared to the two *eat1* genes (Fig. 4c, f). Pyruvate accumulation decreased significantly, indicating that Atf1 was active, but primarily as an esterase/thioesterase as the acetate yields increased. These results show that Eat1 homologues are better catalysts than Sce Atf1 for in vivo ethyl acetate production in *E. coli* BW25113 Δ*ackA*Δ*ldhA* (DE3) under the tested conditions.

The expression of Wan and Kma *eat1* under the control of the LacI/*T7* promoter resulted in 0.2 C-mol_ethyl acetate_/C-mol_glucose_ or higher. However, Wan *eat1* required 10-fold less IPTG to reach the same or higher ethyl acetate yields than Kma *eat1* (Fig. 4ab). Moreover, the strains expressing Wan *eat1* under the control of the XylS/*Pm* promotor produced up to 0.16 ± 0.01 C-mol_ethyl acetate_/C-mol_glucose_ (Fig. 4e), whilst Kma *eat1* produced almost no ethyl acetate (Fig. 4d). This difference may be explained by the fact that the XylS/*Pm* promoter is weaker compared to the LacI/*T7* promoter [2]. The higher yield obtained with lower gene expression levels indicates that Wan Eat1 was more active than its *K. marxianus* homologue under these cultivation conditions.

The ethyl acetate yields increased with rising inducer concentrations (Fig. 4e, f), reached a plateau (Fig. 4a) and even began to decline at higher inducer concentration (Fig. 4b, c). Determining the optimal inducer concentrations thus resulted in significantly improved ethyl acetate yields. For example, optimised IPTG concentrations used for gene induction in *E. coli* BW25113 Δ*ackA*Δ*ldhA* (DE3) (pET26b:hKma Eat1) led to an increase of the ethyl acetate yield from 0.13 ± 0.00 (Fig. 3c) to 0.19 ± 0.00 C-mol_ethyl acetate_/C-mol_glucose_ (Fig. 4a). The highest ethyl acetate yield was achieved in *E. coli* BW25113 Δ*ackA*Δ*ldhA* (DE3) (pET26b:hWan Eat1) that was induced with 0.01 mM IPTG. It produced 0.27 ± 0.01 C-mol_ethyl acetate_/C-mol_glucose_ or 40.7% of the theoretical pathway maximum (Fig. 4b).

Selecting the best AAT gene and optimising, its expression level diminished the metabolic bottleneck present in ethyl acetate production, but pyruvate still accumulated (Fig. 4a–f). This indicated that the conversion efficiency of Eat1 was still insufficient to handle the EMP metabolic pathway flux.

### Using truncated Eat1 variants

**Fig. 5:**
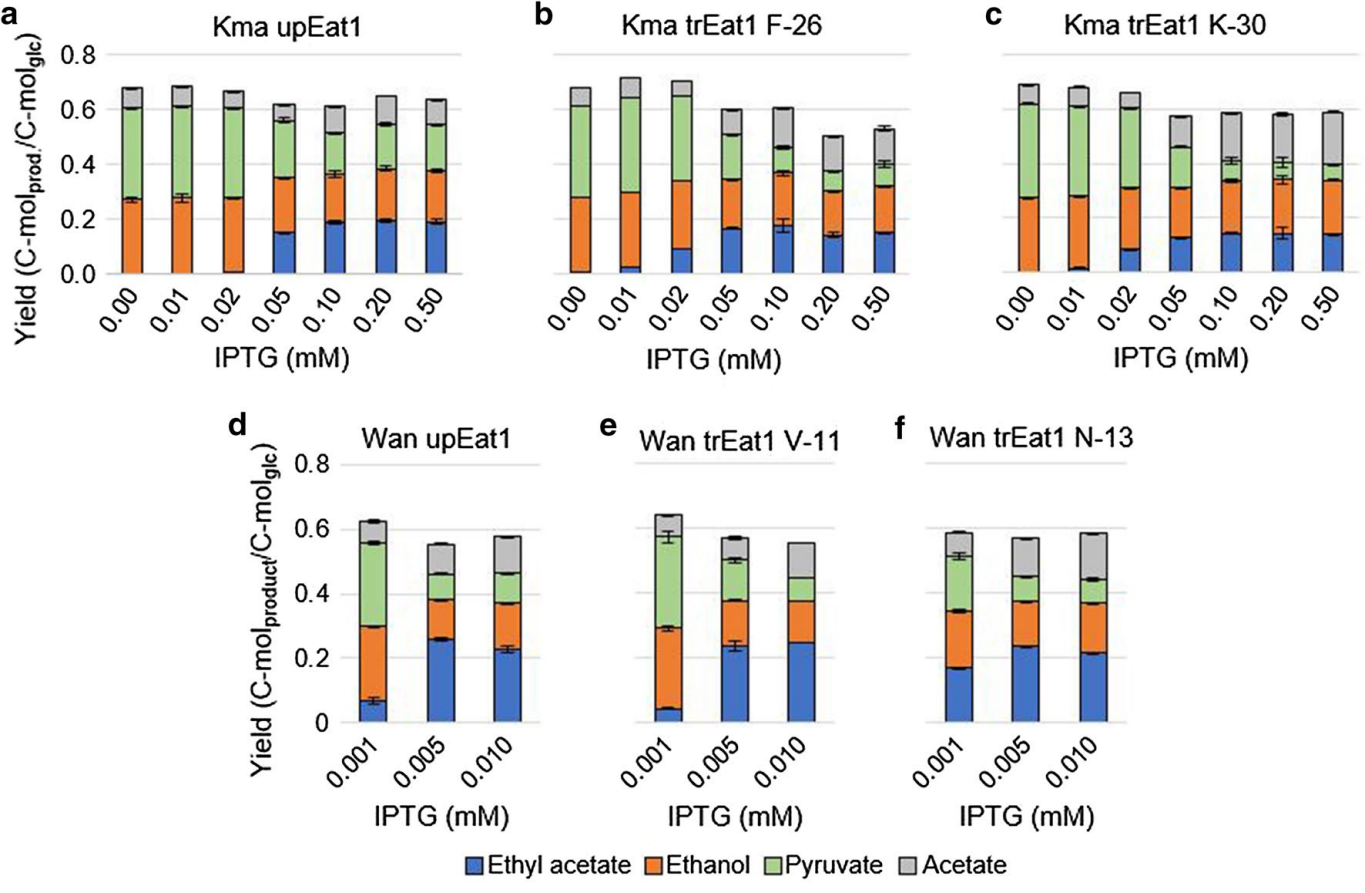
Comparison of truncated eat1 genes under various gene expression levels induced by 0.01–0.5 mM IPTG or 0.001–0.01 mM IPTG. **a**–**c** Fermentation product yields of strains expressing the Kma *Eat1,* the Kma trEat1 F-26 and the Kma trEat1 K-30, respectively, under the control of the LacI/*T7* promoter after 120 h of cultivation. Gene expression was induced with 0.01–0.5 mM IPTG. **d**–**f** Fermentation product yields of strains expressing the Wan Eat1, the Wan *trEat1 V*-*11* and the Wan trEat1 N-13, respectively, under the control of the LacI/*T7* promoter after 120 h of cultivation. Gene expression was induced with 0.001–0.01 mM IPTG. Strains were grown in sealed and N_2_ flushed serum bottles under anaerobic conditions in modified M9 medium at 30 °C and 150 rpm. Genes were expressed in *E. coli* BW25113 Δ*ackA*Δ*ldhA* (DE3) from a series of pET26b plasmids. Succinate and formate were detected but concentrations are not shown. Experiments were performed as biological duplicates; error bars represent the standard deviation. *Kma K. marxianus*, *Wan W. anomalus, Eat1* unprocessed Eat1, *trEat1* truncated Eat1

Removal of the mitochondrial pre-sequences of *K. marxianus* and *W. anomalus* Eat1 resulted in a higher stability of the enzyme when expressed in *E. coli* [20]. We tested if this elevated stability also led to more ethyl acetate production. Optimisation of gene expression levels for Kma trEat1 F-26 and K-30 resulted in a lower accumulation of pyruvate compared to the unprocessed version (Fig. 5a–c), suggesting a higher efficiency of the truncated Eat1 variants. However, this did not result in a higher ethyl acetate yield; only the acetate yield increased.

Nevertheless, the optimum inducer concentration shifted to 0.05 mM IPTG, which was 50% lower compared to the native Eat1. Ethyl acetate production was also higher at 0.01 and 0.02 mM IPTG, indicating that the in vivo production capacity of ethyl acetate improved. At the same time, the acetate yields increased for induction levels above 0.05 mM IPTG, whilst the pyruvate yields decreased (Fig. 5b, c).

At the lowest IPTG concentration, the strains producing the truncated Wan Eat1 (Wan trEat1 N-13) reached a 3.5-fold higher ethyl acetate yield on glucose than the unprocessed Wan Eat1 (Wan Eat1) (Fig. 5e, f). However, at higher IPTG concentrations these differences were absent. The acetate yield in the strain producing Wan trEat1 N-13 also increased relative to the strain producing Wan Eat1. (Figure 5d, f). The increase in acetate production was not as pronounced as with the Kma trEat1 F-26 and K-30 (Fig. 5a–c). No difference was found between the Wan trEat1 V-11 and the unprocessed Wan Eat1 (Fig. 5d, e).

As discussed above, a limiting Eat1 efficiency resulted in the accumulation of ethanol. Pyruvate was produced by *E. coli* BW25113 Δ*ackA*Δ*ldhA* (DE3) to counter the redox imbalance caused by ethanol accumulation. The higher stability of the truncated Eat1 versions did indeed result in a decreased pyruvate yield (Fig. 5b, c, e, f) but the ethyl acetate yield did not increase accordingly. Instead, the acetate yield increased, likely due to the esterase and thioesterase side activities of Eat1.

### Improving ethyl acetate production with H_2_ co-production in controlled bioreactors

In all serum bottle experiments described above, glucose consumption was incomplete, most likely caused by the accumulation of organic acids, especially formate, and the associated pH decreased due to a limited buffering capacity of the medium. To avoid limitations caused by medium acidification, additional cultivations were performed in pH-controlled reactors under anaerobic conditions. To limit the accumulation of formate even further, Na_2_SeO_3_ was added to stimulate the conversion of formate into H_2_ and CO_2_ by Fhl. A constant flow of nitrogen gas was applied to keep the culture conditions anoxic. This resulted in stripping of ethyl acetate, H_2_ and CO_2_ from the broth and the concentrations of these compounds in the exhaust gas were therefore analysed.

**Fig. 6:**
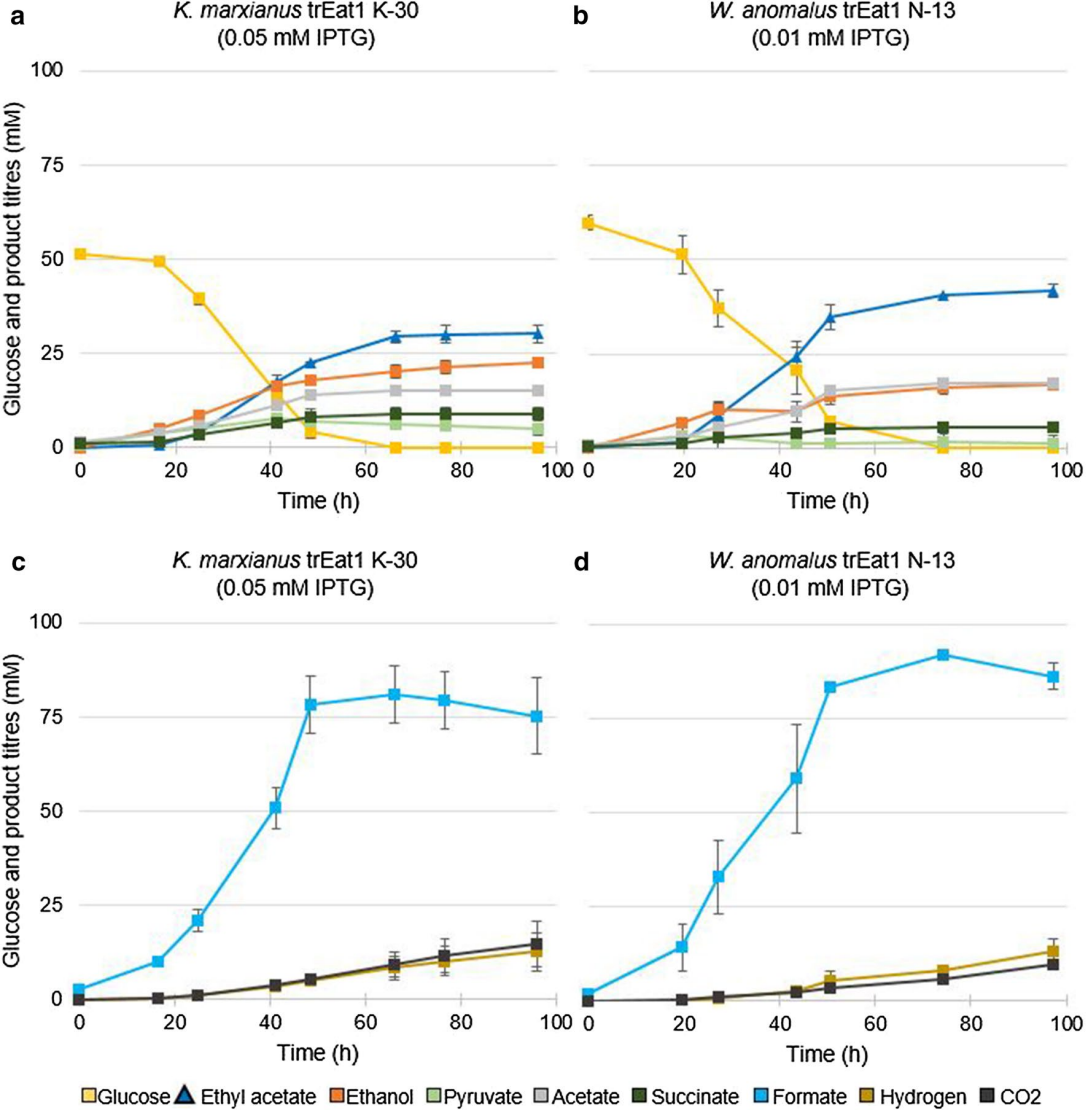
Ethyl acetate production in pH-controlled bioreactors with continuous gas stripping. Two examples of controlled batch fermentations are shown. **a**, **c** Fermentation profile of the strain producing Kma trEat1 K-30 in the presence of 0.05 mM IPTG. **b**, **d** Fermentation profile of the strain producing Wan trEat1 N-13 in the presence of 0.01 mM IPTG. Strains were grown under anaerobic conditions in minimal medium containing 55 mM glucose. Genes were expressed in *E. coli* BW25113 Δ*ackA*Δ*ldhA* (DE3) from a series of pET26b plasmids. The cumulative mass of ethyl acetate, CO_2_ and H_2_ removed by gas stripping was divided by the culture volume of the reactor and in case of ethyl acetate added to concentrations measured in the liquid. Experiments were performed as biological duplicates; error bars represent the standard deviation. *trEat1* truncated Eat1

**Fig. 7:**
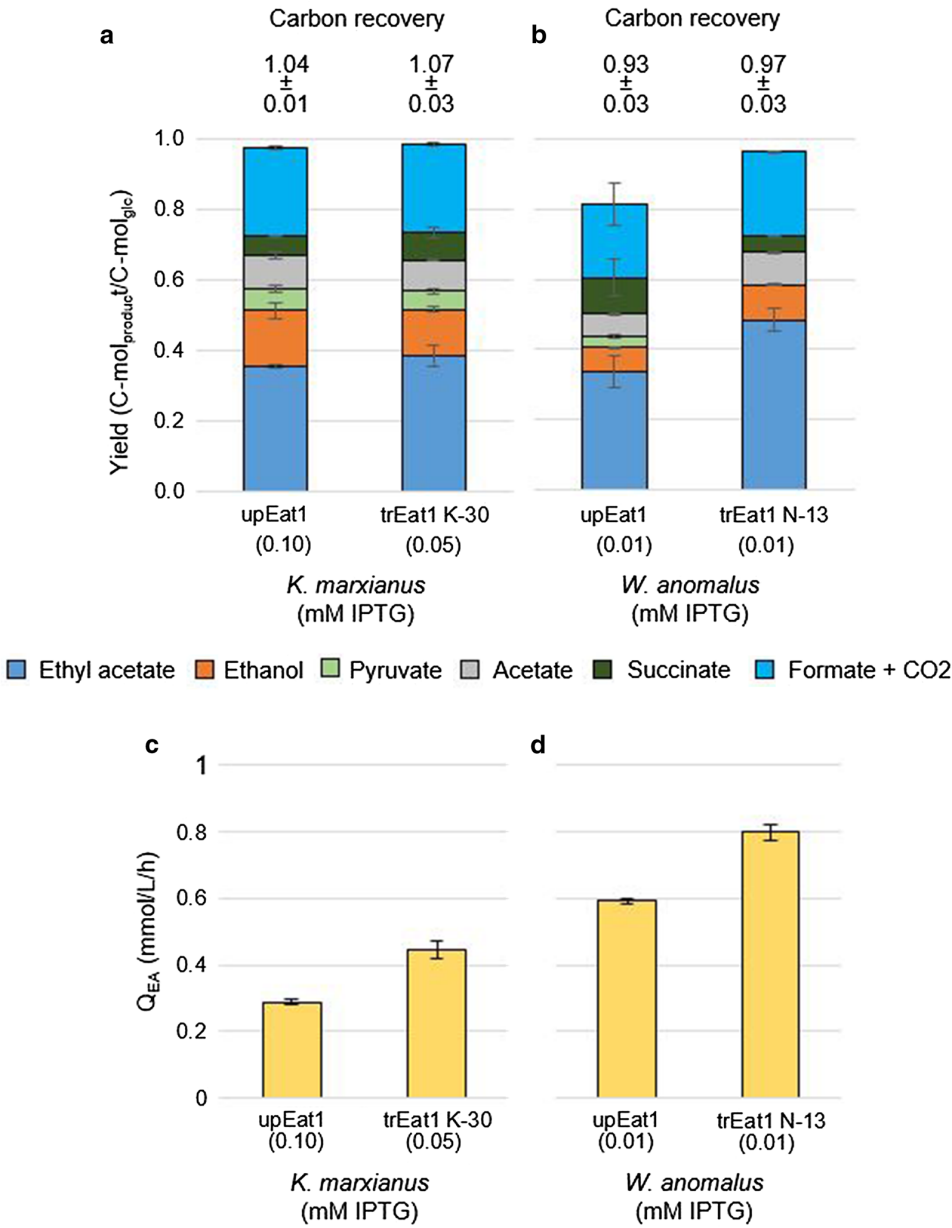
Effect of pH-control and continuous ethyl acetate stripping on product yield and volumetric productivity. **a** Final product yields achieved by cultures producing unprocessed Kma Eat1 and trEat1 K-30 in the presence of 0.05 or 0.1 mM IPTG. The numbers above the bars represent the carbon recovery of the fermentations. **b** Final product yields achieved by cultures producing unprocessed Wan Eat1 and trEat1 N-13 in the presence of 0.01 mM IPTG. The numbers above the bars represent the carbon recovery of the fermentations. **c**, **d** The volumetric productivity of ethyl acetate (Q_EA_) of the fermentation shown in **a** and **b**, respectively. Strains were grown under anaerobic conditions in minimal medium containing 55 mM glucose. Genes were expressed in *E. coli* BW25113 Δ*ackA*Δ*ldhA* (DE3) from a series of pET26b plasmids. The cumulative mass of ethyl acetate, CO_2_ and H_2_ removed by gas stripping was divided by the culture volume of the reactor and in case of ethyl acetate added to concentrations measured in the liquid. Formate and CO_2_ yields were lumped together to compensate for the variation in H_2_ formation. Experiments were performed as biological duplicates or triplicates; error bars represent the standard deviation. *Eat1* unprocessed Eat1, *trEat1* truncated Eat1

We cultivated *E. coli* BW25113 Δ*ackA*Δ*ldhA* (DE3) producing several Eat1 variants. Gene expression was induced with the optimal IPTG concentration of each strain based on the findings of previous experiments (Figs. 4, 5). In contrast to the shake-flask experiments, glucose was fully consumed at the end of the batch fermentations and ethyl acetate production proceeded until glucose was depleted (Fig. 6a, b). Formate was converted into CO_2_ and H_2_ by *E. coli* BW25113 Δ*ackA*Δ*ldhA* (DE3), but conversion percentages were inconsistent and the conversion was incomplete (Fig. 6 c, d). Strains’ conversion was between 6% and 27% of the formate into CO_2_ and H_2_ whilst in most fermentations, the conversion averaged around 10%. There was no correlation between the conversion efficiency, the strain, or reactor vessel. Between 93.0 and 103.7% of the carbon was recovered in all runs performed when biomass formation and the main fermentation products such as ethyl acetate, ethanol, acetate, pyruvate, formate, succinate and CO_2_ were included (Fig. 7a, b).

Consistently, all strains cultivated in pH-controlled bioreactors showed improved performance compared to the serum bottle cultivations. Once the unprocessed Kma Eat1 was induced with an optimal 0.1 mM IPTG, a beneficial effect on ethyl acetate yield was apparent. A yield of 0.35 ± 0.01 C-mol_ethyl acetate_/C-mol_glucose_ corresponded to a 1.8-fold increase when compared to a serum bottle yield of 0.19 ± 0.01 C-mol_ethyl acetate_/C-mol_glucose_, reaching about 50% of the maximum pathway yield. A similar yield was obtained in strains producing Kma trEat1 K-30 in the presence of 0.05 mM IPTG (Fig. 7a).

The best producers tested in pH-controlled bioreactors were *E. coli* BW25113 Δ*ackA*Δ*ldhA* (DE3) producing Kma trEat1 K-30 and Wan trEat N-13. They formed 27.6 ± 3.7 mM (2.4 ± 0.3 g/L) and 42.8 ± 3.3 mM (3.8 ± 0.3 g/L) ethyl acetate from 55.6 ± 2.5 mM (10.0 ± 0.5 g/L) glucose, respectively (Fig. 6, Additional file 1). Generally, ethyl acetate yields were between 1.6- and 2.8-fold higher in bioreactors compared to serum bottles. The highest yield was obtained by the strain producing Wan trEat1 N-13, reaching 0.48 ± 0.03 C-mol_ethyl acetate_/C-mol_glucose_, or 72.3% of the maximum pathway yield.

The yields of ethanol and pyruvate decreased with increasing ethyl acetate yields (Fig. 7a, b). Strains producing the unprocessed Kma Eat1 and Kma trEat1 K-30 in the presence of optimal IPTG concentrations accumulated 66% less pyruvate (Fig. 7a) compared to cultivations in serum bottles (Fig. 5a, c). For the strains producing unprocessed Wan Eat1 and Wan trEat1 N-13 pyruvate accumulation was almost entirely abolished (Figs. 6b and 7b). The ethyl acetate yield for Wan Eat1 was consequently higher compared to the strains producing the Kma Eat1 variants. It should be noted that a statistically significant difference in ethyl acetate yields (*p*  = 0.03) was only found for the strain producing Wan trEat1 N-13 (Fig. 7a, b). This strain converted approximately 72% of glucose to ethyl acetate based on the maximum pathway yield.

Not only did the trEat1 variants require lower induction levels and accumulated less by-products, glucose was also depleted faster. As a result, the volumetric productivity of ethyl acetate (Q_EA_) was higher. The Q_EA_ of the strain producing Kma trEat K-30 (0.05 mM IPTG) was 35% higher (*p* = 0.013) compared to the strain producing unprocessed Kma Eat1 (0.1 mM IPTG) (Fig. 7c). A similar trend was present in *E. coli* BW25113 Δ*ackA*Δ*ldhA* (DE3) producing unprocessed Wan Eat1 and trEat1 N-13 in the presence of 0.01 mM IPTG. The Q_EA_ of the latter strain was 26% higher (*p*-value = 0.042) compared to the strain producing the unprocessed Wan Eat1 (Fig. 7d).

The hydrolysis of ethyl acetate by the side activity of Eat1 might be restricted by efficiently removing all ethyl acetate by gas stripping. But due to low gas flow rates, ethyl acetate still accumulated in the liquid during the fermentation. At times of maximum productivities, liquid ethyl acetate concentrations ranged from 2.87 ± 0.1 mM for Kma Eat1 with 0.05 mM IPTG induction to up to 14.7 ± 0.4 mM for Wan trEat1 N-13 with 0.01 mM IPTG induction (data not shown).

## Discussion

We describe the engineering of *E. coli* for the anaerobic production of ethyl acetate and the different optimisation efforts to further improve the product yield. In all cultivations of the metabolically streamlined *E. coli* BW25113 *ΔackAΔldhA* (DE3) (pET26b:hKmaEat1) substantial amounts of ethanol and pyruvate were formed, in addition to ethyl acetate. This redox-neutral accumulation of pyruvate and ethanol indicated that the in vivo activity of Eat1 was insufficient to cope with the supply of acetyl-CoA and ethanol.

Screening of *E. coli* strains expressing three different AATs revealed that their capacity to produce ethyl acetate under anaerobic conditions varied significantly. The expression of Sce *atf1* evoked acetate production, which may be related to its thioesterase activity [35]. Alternatively, Atf1 may act as an esterase, but this has not been determined. It is unknown whether ethanol inhibits the hydrolytic activity of Sce Atf1 in the same way as was demonstrated for Eat1 [17]. It was observed before that Atf1 exhibits low affinity for the catalysis of ethyl acetate despite external ethanol addition [15]. On the other hand, Sce Atf1 enabled isobutyl acetate production at 80% of the pathway maximum [38], which indicates that it can be an effective AAT in *E. coli*. Thus, the inefficient ethyl acetate production by Sce Atf1 may have been caused by differences in substrate specificity or fermentation conditions. However, Atf1 in *S. cerevisiae* is most active under anaerobic conditions due to higher gene expression [12], suggesting that anoxic conditions should not be a bottleneck in Atf1 activity. Nevertheless, the results of this study disqualified it as catalyst for effective ethyl acetate production under the tested conditions.

Next to plasmid maintenance also inducer compounds are commonly imposing an additional burden to the cells [7, 31]. Whilst the LacI/*T7* promoter system is widely applied in molecular engineering studies, it is known to have a strong expression as well as exhibiting some leaky behaviour under non-induced conditions [41]. Moreover, inclusion bodies may form if translation rates are too high and can been a bottleneck in the heterologous expression of AATs [53, 56]. It is possible that lower IPTG concentrations increased the amount of correctly folded protein and led to higher ethyl acetate production. In contrast, the XylS/*Pm* promoter system is weaker, but remarkably tight and titratable [2]. In the present case, however, the strong LacI/*T7* promoter system more efficiently triggered *eat1* activity and ethyl acetate production. Only for Wan Eat1, ethyl acetate formation was observed with the XylS/*Pm* system. This may result from an overall higher efficiency of the Wan Eat1 variant, compared to Kma Eat1.

Consistently, Wan Eat1 and its truncated variant were most efficient. Strains producing Wan Eat1 variants formed up to 15% more ethyl acetate in vivo compared to strains producing Kma Eat1. Optimising the Eat1 efficiency by selecting a better Eat1 variant and improving the expression indeed led to a significant decrease in pyruvate accumulation. Performing similar optimisations on truncated variants, had similar effects, but interestingly also led to lower induction levels for similar or better results. Manual cleavage of the N-termini affected the enzymes’ cellular localisation in yeasts and diminished or enhanced catalytic performance in *E. coli*, emphasising the importance of those pre-sequences [20, 25]. Proper cleavage likely improved protein stability, which was reflected by the lower required inducer concentration.

Whilst especially the transfer to pH-controlled reactor systems boosted general performance of the presented ethyl acetate production process, production of other dissimilatory products, like succinate, ethanol and acetate, needs to be further minimised. As fermentations were performed under anaerobic conditions, ethanol and acetate could not be assimilated for additional ethyl acetate formation but remained as by-products of the fermentation. The disruption of *ackA* did not block acetate synthesis completely. The predominant acetate-forming route under anaerobic conditions is the conversion of acetyl-CoA to acetyl-P and further to acetate [52]. Two genes are involved in this pathway, phosphotransacetylase (*pta*) and acetate kinase (*ackA)*, whilst other enzymes with similar catalytic activities, such as propionate kinase are able to perform the same reaction [14]. Disrupting *pta* and additional acid kinases might block acetate production completely.

Acetate accumulation by the *ackA* knockout strain, and to some extent ethanol accumulation, may also result from the hydrolytic side activities of Eat1. It has been shown that this esterase and thioesterase activity is prevented above a critical ethanol concentration [17]. Below this critical concentration, there was no net ethyl acetate synthesis and ethanol and acetate were produced instead. Under the tested conditions, Atf1 exhibited more esterase and thioesterase activities, barely producing ethyl acetate. The tested eat1 variants of *W. anomalus* and *K. marxianus* also showed increased acetate levels at higher induction levels. Better understanding of the protein structure and catalytic mechanisms is needed to streamline the desired catalytic activities even further.

A build-up of high formate levels could be detrimental to cell growth and function and might have inhibited the serum bottle fermentations [49]. In batch bioreactors, this problem might be tackled by applying pH control. Moreover, converting formate to CO_2_ and H_2_ via the Fhl complex would allow for co-production of ethyl acetate and H_2_, the latter also being a valuable biofuel [5]. In our experiments, however, formate was only partially converted (between 6% and 27%), which was also experienced in other studies [37, 54]. The reason for the high variability is not clear, but it may be due to the complex transcriptional regulation of the 15 genes that are required to form an active Fhl complex [57, 3, 39]. The issue might be prevented in the future by constitutively overexpressing *fhlA*, the transcriptional activator of the Fhl system to improve H_2_ production [39, 55]. Addition of nickel may also be explored as this compound is required in the functional Fhl system [32].

In situ product removal via gas stripping has already been applied in some yeast production systems [43, 29]. Primarily, it improves downstream processing or can be used to prevent product inhibition during fermentations [45, 19]. Whilst no critical concentrations of ethyl acetate were reached in the performed fermentations, gas stripping could benefit the fermentations by limiting ethyl acetate hydrolysis. However, temporary accumulation and hydrolysis of ethyl acetate in the medium could not be avoided by the applied stripping rates, particularly for efficient ethyl acetate producers such as Kma trEat1 K-30 or Wan trEat1 N-13. Therefore, performances of the respective strains may still improve when higher stripping rates are applied.

Whilst the reduction of degradation of the product by gas stripping improves the performance in the current research, it primarily aims at preventing product toxicity [19]. Currently the reached titres of ethyl acetate are well below toxic levels for *E. coli* [51] but higher inoculation densities and the switch to fed-batch systems should benefit the final product titres. Whether the volumetric productivities can also compete with those reached by aerobic systems, is another factor that needs to be evaluated in the future.

The yield of 72% of the maximum pathway did already exceed the best ethyl acetate yield reported for *K. marxianus* converting whey sugars (predominantly lactose) to ethyl acetate under aerobic conditions, reaching 56% of the theoretical maximum pathway yield [45].

## Conclusion

We demonstrated that *E. coli* can be engineered to efficiently convert glucose to ethyl acetate as the primary fermentation product, which may serve as a point of reference for future development of biobased ethyl acetate-production processes in which Eat1 serves as the AAT catalyst. The combined effects of several rounds of metabolic, protein and process engineering resulted in an up to 14.3-fold increase in ethyl acetate yield. The highest ethyl acetate yield was achieved with *E. coli* BW25113 Δ*ackA*Δ*ldhA* (DE3) producing Wan trEat1 N-13 in the presence of 0.01 mM IPTG. This strain formed 0.49 ± 0.03 C-mol_ethyl acetate_/C-mol_glucose_, which corresponds to ~ 72% of the theoretical pathway maximum.

## Materials and methods

### Strain and plasmid construction

The strains and plasmids used in this study are listed in Tables 1 and 2, respectively. The pET26b-XylS/*Pm* plasmids were obtained by replacing the lacI/*T7* promoter of pET26b with the XylS/*Pm* promoter [2] using 2X HiFi assembly master mix (NEB) according to the supplier protocol. All *K. marxianus* and *W. anomalus eat1* genes were cloned with a Strep-tag or 6-His-tag, respectively, to facilitate protein purification. PCR amplifications were performed with Q5 polymerase (NEB) according to supplier instructions.

**Table 1:** Strains used in this study

Strain	Characteristics	Source
*Escherichia coli* BW25113 (DE3)	Wild type with integrated DE3 lysogen	[48]
*Escherichia coli* BW25113 Δ*ackA*Δ*ldhA*	Disruption of lactate and acetate production (via *ackA*)	[20]
*Escherichia coli* T7 Express	fhuA2 [lon] ompT gal (λ DE3) [dcm] ∆hsdS λ DE3 = λ sBamHIo ∆EcoRI-B int::(LacI::PlacUV5::T7 gene1) i21 ∆nin5	NEB
*Escherichia coli* NEB^®^ 5-alpha	*fhuA2 Δ(argF*-*lacZ)U169 phoA glnV44 Φ80 Δ(lacZ)M15 gyrA96 recA1 relA1 endA1 thi*-*1 hsdR17*	NEB

**Table 2:** Plasmids used in this study

Plasmid	Promoter	Gene/Protein	Source
pET26b	LacI/*T7*	/	This study
pET26b:hWanEat1	LacI/*T7*	Codon harmonised *eat1* from *Wickerhamomyces anomalus* DSM 6766	[17]
pET26b:hKmaEat1	LacI/*T7*	Codon harmonised *eat1* from *Kluyveromyces marxianus* DSM 5422	[20]
pET26b:opSceAtf1	LacI/*T7*	Codon optimised atf1 from *Saccharomyces cerevisiae* [38]	This study
pET26b: XylS/*Pm* -hWanEat1	XylS/*Pm*	Codon harmonised *eat1* from *Wickerhamomyces anomalus* DSM 6766	This study
pET26b: XylS/*Pm* hKmaEat1	XylS/*Pm*	Codon harmonised *eat1* from *Kluyveromyces marxianus* DSM 5422	This study
pET26b: XylS/*Pm* opSceAtf1	XylS/*Pm*	Codon optimised atf1 from *Saccharomyces cerevisiae*	This study
pET26b:hKma trEat1 F-26	LacI/*T7*	Kma Eat1 truncated at F-26	[20]
pET26b:hKma-trEat1-K30	LacI/*T7*	Kma Eat1 truncated at K-30	[20]
pET26b:hWan-trEat1-V11	LacI/*T7*	Wan Eat1 truncated at V-11	[20]
pET26b:hWan-trEat1-N13	LacI/*T7*	Wan Eat1 truncated at N-13	[20]

### Cultivation

Routinely*, E. coli* strains were grown on LB medium supplemented with kanamycin (50 μg/mL) or spectinomycin (50 μg/mL). Sterile 250-mL serum bottles were filled with 50 mL modified M9 medium, consisting of M9 salts (Difco, 1X), glucose (55 mM), MgSO_4_ (2 mM), CaCl_2_ * 2 H_2_O (0.1 mM), MOPS (100 mM) and 1 mL 1000X trace elements and vitamins each according to Verduyn et al. [46], and used for anaerobic cultivation experiments. The serum bottles were made anaerobic by flushing with nitrogen. For precultures single colonies were transferred to 10 mL LB medium in a 50-mL tube and grown overnight at 30 °C and 250 rpm. A second overnight cultivation under same conditions was performed after 1–2 mL of the LB culture was transferred to 50 mL modified M9 medium in a 250-mL Erlenmeyer flask. The anaerobic serum bottles were inoculated to an initial OD of 0.2 and incubated at 30 °C and 150 rpm. The inducing reagents isopropyl β-d-1-thiogalactopyranoside (IPTG) (0.01–0.5 mM) or m-toluate (0.021 mM) were added to induce gene expression when appropriate. Experiments were performed as biological duplicates. Ethyl acetate production in serum bottles was measured only in the liquid phase.

### Batch fermentations

Anaerobic fermentations were performed in 1.5-L bioreactors (Applikon) in 0.5 L defined medium. The fermentation medium contained glucose (55 mM), (NH4)_2_SO_4_ (37.8 mM), KH_2_PO_4_ (22 mM), NaCl (171 mM), kanamycin (100 µg/mL) and Na_2_SeO_3_ (0.3 mg/L) to promote hydrogen formation, unless mentioned otherwise. The medium was supplemented with vitamins and trace elements [46]. The fermentation broth was stirred at 400 rpm with a Rushton turbine controlled by an ADI 1012 Motor Controller (Applikon). pH was kept constant at 7.0 by automatic addition of 3 M KOH or 0.5 M H_2_SO_4_. The temperature was controlled at 30 °C by a Thermo Circulator ADI 1018 (Applikon). Anaerobic conditions were maintained using oxygen-impermeable Marprene tubing (Watson-Marlow) and constant sparging with 3 L/h N_2_. Inocula were prepared by transferring 0.5 mL fresh overnight LB pre-culture to 50 mL modified M9 medium in a 250-mL Erlenmeyer flask. The culture was grown overnight aerobically at 30 °C and 250 rpm. The reactors were inoculated to an initial OD_600_ of 0.4. Metabolites in the liquid phase were measured by high-performance liquid chromatography (HPLC) and gas chromatography coupled to a flame ionisation detector (GC-FID). Online measurements of volatile compounds and gases removed from the vessel by gas stripping were performed with a δB Process Mass Spectrometer (MS, Thermo Scientific™).

### Calculations

The gaseous concentration of ethyl acetate, CO_2_ and H_2_ (C_C, gas_, mol/L) in the outflow was calculated based on the ideal gas law according to Eq. 1 follows:

1 $$C_{C,gas} = \frac{{\frac{{X_{C, gas} }}{{P_{atm} }}}}{R*T}.$$

With C representing the corresponding compound (ethyl acetate, CO_2_ and H_2_), X_C, gas_ the volumetric fraction of compound C in the gas (-), P_atm_ the atmospheric pressure (Pa), R the ideal gas constant (m^3^ Pa/mol/K) and T the temperature (K). The cumulative mass of compound C (m_C,gas_, mol) stripped up to each time point (t_n_, h) was calculated using Eq. 2.

2 $$m_{C,gas} \left( {t_{n} } \right) = \frac{{C_{C,gas} \left( {t_{n - 1} } \right) + C_{C,gas} \left( {t_{n} } \right)}}{2}* \frac{{F_{gas,out} \left( {t_{n - 1} } \right) + F_{gas,out} \left( {t_{n} } \right)}}{2}* \Delta t + m_{C,gas} \left( {t_{n - 1} } \right)$$

where the average gaseous concentration of the product between time points *t*_n-1_ and *t*_n_ is calculated from Eq. 1 (mol/L), *F*_gas,out_ is the total volumetric gas flow rate leaving the reactor (L/h), Δt is the time between two time points (h) and *m*_C,gas_(t_n-1_) is the amount of compound C stripped up to the previous time point (mol). *F*_gas,out_ was calculated assuming *N*_2_ as an inert gas and knowing the total volumetric gas flow into the reactor (*F*_gas,in_) and the volumetric fractions of *N*_2_ in the corresponding in- and outflows (*X*_N2,in_, *X*_N2,out_) at time point *t* using Eq. 3.

3 $$F_{gas,out} = \frac{{X_{N2,in} }}{{X_{N2,out} }}* F_{gas,in}$$

The cumulative mass of stripped ethyl acetate after Eq. 3 was divided by the culture volume in the bioreactor and added to the current ethyl acetate concentration in the liquid. The resulting value is an apparent ester concentration at time *t*_n_ which would be found in the culture medium if no stripping was applied.

### Carbon balance calculation

Carbon balances were calculated according to Eq. 4.

4 $$C_{balance} = \frac{{{\text{C}} - {\text{mol products formed}}}}{{{\text{C}} - {\text{mol glucose consumed}}}}$$

The compounds included in the calculation were glucose, ethyl acetate, ethanol, acetate, succinate, pyruvate, formate and CO_2_. Biomass formation was included in the calculation assuming a biomass composition of CH_2_O_0.5_N_0.2_ and an experimentally determined conversion factor of 0.3232 from OD_600_ to g/L dry weight (data not shown).

### Analytical

Glucose and organic acids were analysed by HPLC on an Agilent 1290 LC II system, equipped with an Agilent 1290 Infinity Binary Pump, Agilent 1290 Infinity Autosampler, Agilent 1290 Infinity diode array detector operated at 210 nm, and an Agilent 1260 Infinity RI detector operated at 45 °C. Either an Aminex HPX-87H (Bio-Rad) or a Rezex ROA-Organic Acid H + (Phenomenex) column was used with a mobile phase of 0.008 mM H_2_SO_4_. The HPLC was operated at 0.8 mL/min and 60 °C. Propionic acid (50 mM) was used as an internal standard.

Ethyl acetate and ethanol in liquid samples were measured by an Agilent 7890B gas chromatograph equipped with a flame ionisation detector (GC-FID) and an Agilent 7693 autosampler. Samples were analysed by injecting 0.5 μL of liquid sample onto a Nukol^TM^ column (30 m × 0.53 mm, 1.0 μm coating, Supelco). The column temperature was maintained at 50 °C for 2 min and increased to 200 °C at a rate of 50 °C/min. The split ratio was 10. 1-Butanol (2 mM) was used as an internal standard.

## Supplementary information

**Additional file 1: Table S1.** Overview of pH-controlled batch fermentations in 1.5L Applikon bioreactors with continuous gas stripping. Measured and calculated concentrations of main fermentation products, carbon balance and C-mol yields at end of fermentations are represented as average (AV) with standard deviations (SD) for each duplicate. *E. coli* BW25113 Δ*ackA*Δ*ldhA* (DE3) producing Eat1 variants from pET26b plasmids were grown under anoxic conditions in minimal medium containing 55 mM glucose. Expression of Eat1 was induced by IPTG.

## Data Availability

All data generated or analysed during this study are included in this published article and its additional information files.

## Abbreviations

AA: Amino acid
AAT: Alcohol acetyl transferase
Adh: Alcohol/aldehyde dehydrogenase
AckA: Acetate kinase
Acs: Acetyl-CoA synthetase
Ald: Acetaldehyde dehydrogenase
Atf1: Alcohol acetyltransferase 1
C_C, gas_: Gaseous concentration of compound *C* (mol/L)
EA: Ethyl acetate
Eat1: Ethanol acetyltransferase 1
EMP: Embden–Meyerhof–Parnas
*F*_gas,in_: Total volumetric gas flow into the reactor (L/h)
*F*_gas,out_: Total volumetric gas flow rate leaving the reactor (L/h)
Fhl: Formate hydrogen lyase
IPTG: Isopropyl β-d-1-thiogalactopyranoside
Kma: *Kluyveromyces marxianus*
Ldh: Lactate dehydrogenase
*m*_C,gas_: (cumulative) Molar mass of compound *C* in gas phase (mol)
*P*_atm_: Atmospheric pressure (Pa)
Pdc: Pyruvate decarboxylase
Pfl: Pyruvate formate lyase
Pr: Fermentation products
Pta: Phosphotransacetylase
Q_EA_: Volumetric productivity of ethyl acetate (mmol/L/h)
R: Ideal gas constant (8.314 J/mol/K)
Sce: *Saccharomyces cerevisiae*
T: Temperature (K)
*t*_n_: Time point (h)
trEat1: Truncated Eat1
Wan: *Wickerhamomyces anomalus*
*X*_C, gas_: Volumetric fraction of compound *C* in the gas (L/L)
