# Supplementary material for: Multilevel optimisation of anaerobic ethyl acetate production in engineered Escherichia coli
Source: Biotechnol Biofuels. 2020 Apr 7;13:65. doi: 10.1186/s13068-020-01703-1 (PMC7137189; doi:10.1186/s13068-020-01703-1)
Supplement: Supplementary file 1 — Additional file 1: Table S1. Overview of pH-controlled batch fermentations in 1.5L Applikon bioreactors with continuous gas stripping. Measured and calculated concentrations of main fermentation products, carbon balance and C-mol yields at end of fermentations are represented as average (AV) with standard deviations (SD) for each duplicate. E. coli BW25113 ΔackAΔldhA (DE3) producing Eat1 variants from pET26b plasmids were grown under anoxic conditions in minimal medium containing 55 mM glucose. Expression of Eat1 was induced by IPTG. [file 13068_2020_1703_MOESM1_ESM.docx]

# Additional file

Supplementary Table 1: Overview of pH-controlled batch fermentations in 1.5L Applikon bioreactors with continuous gas stripping. Measured and calculated concentrations of main fermentation products, carbon balance and C-mol yields at end of fermentations are represented as average (AV) with standard deviations (SD) for each duplicate. *E. coli* BW25113 Δ*ackA*Δ*ldhA* (DE3) producing Eat1 variants from pET26b plasmids were grown under anoxic conditions in minimal medium containing 55 mM glucose. Expression of Eat1 was induced by IPTG.

|  |  | Kma Eat1 | | Kma trEat1 K30 | | Wan Eat1 | | Wan trEat1 N13 | |
| --- | --- | --- | --- | --- | --- | --- | --- | --- | --- |
|  | Unit | AV | SD | AV | SD | AV | SD | AV | SD |
| Ethyl acetate | mM | 27.8 | 0.6 | 29.5 | 1.7 | 27.6 | 3.7 | 42.8 | 3.3 |
| Ethanol | mM | 25.3 | 2.6 | 20.3 | 1.7 | 11.7 | 0.0 | 17.7 | 0.4 |
| Pyruvate | mM | 6.6 | 0.7 | 5.5 | 0.9 | 3.2 | 0.4 | 0.0 | NA |
| Acetate | mM | 14.8 | 1.1 | 13.5 | 0.9 | 10.4 | 0.7 | 16.1 | 0.3 |
| Lactate | mM | 1.0 | 0.1 | 1.0 | 0.1 | 0.6 | 0.1 | 1.4 | 0.0 |
| Succinate | mM | 5.7 | 1.2 | 8.0 | 1.2 | 11.5 | 6.0 | 5.6 | 0.1 |
| Formate+CO_2_ | mM | 79.5 | 1.2 | 77.4 | 3.1 | 68.4 | 19.7 | 84.0 | 0.2 |
| Carbon balance | - | 1.04 | 0.05 | 1.07 | 0.03 | 0.93 | 0.03 | 0.97 | 0.03 |
| Yield EA/Glc | Cmol/Cmol | 0.35 | 0.01 | 0.38 | 0.03 | 0.34 | 0.05 | 0.48 | 0.03 |
| Yield EtOH/Glc | Cmol/Cmol | 0.16 | 0.02 | 0.13 | 0.01 | 0.07 | 0.00 | 0.10 | 0.00 |
| Yield Pyr/Glc | Cmol/Cmol | 0.06 | 0.01 | 0.05 | 0.01 | 0.03 | 0.00 | 0.00 | NA |
| Yield Ac/Glc | Cmol/Cmol | 0.09 | 0.01 | 0.09 | 0.00 | 0.06 | 0.00 | 0.50 | 0.00 |
| Yield Suc/Glc | Cmol/Cmol | 0.05 | 0.00 | 0.08 | 0.01 | 0.11 | 0.05 | 0.05 | 0.00 |
| Yield For+CO_2_/Glc | Cmol/Cmol | 0.25 | 0.01 | 0.25 | 0.00 | 0.21 | 0.06 | 0.24 | 0.00 |
